# 

**Published:** 1890-12-06

**Authors:** 


### NOTICE TO CORRESPONDENTS.

All MS., Letters, Books for Review, and other matters intended for the Editor should be addressed THE EDITOR, THE LODGE, PORCHESTER SQUARE, LONDON, W.

The Editor cannot undertake to return rejected M.S., even when accompanied by stamped directed envelope.

**Notice to Advertisers and Subscribers.**

All Advertisements, Orders for Copies of THE HOSPITAL, and Business Communications should be addressed to The Publisher, *not to the Editor*, THE HOSPITAL, 140, Strand, W.C.

**Bound Volumes of "The Hospital."**

Vol. VI., containing full page portraits of T.R.H. the Prince and Princess of Wales, and Miss Florence Nightingale, is still obtainable.

Vol. VII. and all the earlier volumes are also for sale.

Orders will be received for Vol. VIII., 4s. post free. Cases for binding may be had of the Publisher, at 1s. 6d. each.

**To Residents, Secretaries, and Matrons.**

The Editor will be glad to have the Regulations and each Report as issued by Asylums, Hospitals, Convalescent and Nursing Institutions, as well as those of other Charities, and an early copy in duplicate of all Appeals and Festival Notices, etc., addressed to *The Editor, The Lodge, Porchester Square, London, W.* Any superintendent, secretary, matron, or other chief official who regularly sends such information may be registered, on application to the Publisher, to receive free of cost a cover for binding THE HOSPITAL in half-yearly volumes.

### BURDETT'S HOSPITAL ANNUAL FOR 1890

**May be obtained of the Publisher, THE HOSPITAL Offices, 140, Strand, W.C., price 2s. 6d. limp boards, or 3s. 6d. cloth. All orders must be accompanied by a remittance.**

### SCRAPS AND GLEANINGS.

CHESTER is suffering from an epidemic of measles.

A "PEOPLE'S PALACE" has been opened at Helsingfors.

IRVINE has subscribed £62 to Glasgow Wastern Infirmary.

A SITE has been chosen at Birkenhead for a new fever hospital.

ALEXANDRE JACQUES has commenced a 47 days 'fast at Christiana.

DR. NORMAN KERR writes in the *New Review* on "Ether Drinking."

THE cold weather has lowered the death-rate from diphtheria and typhoid.

The Princess of Wales sent a large supply of bon-bons to the lepers of Robben Island.

DR. EDWARD BERDOE writes in the *Fortnightly Review* on "Dr. Koch's Consumption Cure."

THE Kyrle Society gave its first concert for the season at Greenwich Workhouse on November 20th.

SALOP INFIRMARY says its stimulant bill is high, owing to the many cases of influenza treated during the year.

THE District Inspector has called attention to the want of proper hospital accommodation to Stockport Workhouse.

THE Board of Management of Bromwich Hospital, have issued an appeal for £5,000 to enable them to enlarge the hospital.

AT Manchester Royal Infirmary a woman, aged 31, who was bitten by a terrier about a month ago, has died of hydrophobia.

THE late Mr. T. E. Ryan bequeathed the munificent sum of £10,000 to the funds of the Royal Hospital for Incurables, Dublin.

MRS. GARRETT ANDERSON last week opened a bazaar in aid of supporting two cots in the North-Eastern Hospital for Children.

THE committee at Guy's, who are preparing the new edition of the Pharmacopœia, have issued a blank for "Suggestions" to all old Guy's men.

A CHEMIST at Liverpool has in his shop a basket for the collection of books and magazines for the Kyrle Society to distribute amongst hospitals.

THE recent decision in the Wheeldon case has created some alarm among the chemists and druggists regarding the position of unregistered assistants.

MISS CURZON, of Nottingham, has left £500 to the Women's Hospital, £200 to the General Hospital, £106 to the Children's Hospital, and other sums to other charities.

DR. EDWARD DOUGLAS, practising at Morpeth, was found dead in bed on November 25th. He was the son of Mr. David Douglas, the well-known Edinburgh publisher.

THERE are over 17,000 lepers in Bengal, 13,000 in Madras, and 13,000 in Bombay. Certainly the Indian Government ought to try and secure the decrease of this terrible disease.

A CONSUMERS' LEAGUE has been founded, which will publish a list of those shops which do not sweat their workers. Consumers will be asked to deal only at shops on this list.

A MEMORIAL bust of the late Dr. Matthews Duncan will be placed in Aberdeen University, and it is also intended to establish a memorial prize or scholarship in connection with the university medical school.

THE *St. James's Gazette* is informed that by 73 votes against 29 the Charity Organization Society has decided that General Booth's scheme is, in its opinion, impracticable and not likely to produce beneficial results.

THE staff of the Dental Hospital of London, with the past and present students of that institution, had their annual dinner on Saturday at the Holborn Restaurant. Dr. Joseph Walker presided, and about 100 gentlemen were present.

THE annual meeting of the Society for the Prevention of Cruelty to Children was held on Saturday at the Victoria Hall, Ealing. Mr. Waugh was at his post as usual, though Lord George Hamilton was unfortunately prevented from occupying the chair.

A MEETING of the council of the Hospital Sunday Fund was held last week at the Mansion House. According to the annual report the collection exceeded every former record by more than £1,000, the total amount collected being £42,814, as against £41,744 in 1889.

AT a quarterly general court of the governors of the Middlesex Hospital, Lord Rothschild was unanimously appointed a vice-president of the hospital, in place of the late Sir Richard Wallace. The amount collected from the alms-boxes outside the hospital during the past year was £42, and from the boxes inside a little over £9.

### BOOKS RECEIVED.

ALLEN, W. H., AND CO.

"Lessons on Health." Arthur Newsholme, M.D.

BELL AND SONS.
"Pasteur and Rabies." T. M. Dolan, M.D.

CASSELL AND CO.
"The Colonist's Medical Handbook." E. A. Barton, M.R.C.S. (2s. 6d.)

CHURCHILL, J. AND A.
"The Dignity of Woman's Health; and the Nemesis of its Neglect." R. R. Rentoul, M.D. (3s. 6d.)

LEWIS, H. K.
"A Text Book of the Diseases of the Ear." By Dr. J. Gruber. Translated from the second German edition, and edited by Edw. Law, M.D., and Coleman Jewell, M.B. (Lond.).

LIVINGSTONE, E. AND S.
*Catechism Series*: "Public Health: Water"; and "Public Health: Air and Ventilation."

MULLAN AND SONS, W. (BELFAST.)
"Manual of Urine Testing." Edited by John Scott, B.A. (1s.)

### The Student of Medicine.

[All communications for this Supplement should be addressed to The Editor of The Hospital, at 140, Strand, with the words, "Students' Column," written in the left hand top corner of the envelope.]

### EDITORIAL NOTES.

The Bogie Man.—This is the title of the popular song of the day. In the superstitious ages, and still among the savage races, various forms of phantasms have been held in great respect, and often with much horror by the people. It is very interesting to see how in many cases some real or imagined form, or more frequently an idea, has completely ruled large numbers of people, both in the smaller things of everyday life and also in the greater crises, not only of individuals, but also of whole tribes or nations. The word "bogie" is said to come from "Zern-bogus," the God of Evil, of Saxon origin, and the abbreviation of this gives the word as we find it in the present day. The threat of a visit from this mysterious personage is used in the nursery to deter the children from acts of naughtiness, as determined by the nurse or mother. As the child grows up the word becomes changed, and the individual is now warned from anti-social actions by a future interview with a similar mysterious being. Many people, even of some education, and among civilized nations, at the present day have their own ideal God of Evil, and many pay much respect to his deterring voice in their actions through life. The students of medicine of the present day in London appear to have their "bogie-man," but he has taken a more material shape, and is generally known as the examiner, and is held by many not necessarily idle students in much unwholesome dread. The thought of such a one causes many sleepless nights and troubled dreams, and the face of infallibility, as assumed by some examiners, is ever before their mind's eye. It is very painful to have to believe that the examiner of the day should be looked upon as an awful judge, one who is ever eager to catch and trip the unfortunate students, many of whom become so completely demoralized by the prolonged mental anguish that precedes the examination that, however well they may know their work, they at once appear in the light of ignorant fools, before the eyes of an infallible official.

### APPOINTMENTS.

At St Thomas's Hospital: G. E. Hanson, M.D., B.C., Cantab, L.R.C.P., M.R.C.S., has been appointed clinical assistant in the skin department; H. C. Bristowe, M.B.Lond., L.R.C.P., M.R.C.S., has been reappointed clinical assistant in the eye department; H. J. Carstairs, L.R.C.P., M.R.C.S., has been appointed clinical assistant in the throat department; H. J. Cooper, M.A., M.B., B.C.Cantab, L.R.C.P., M.R.C.S., has been reappointed non-resident house physician; F. E. Forward, L.R.C.P., M.R.C.S., has been appointed clinical assistant in the skin department; W. S. Griffith, M.A.Cantab, L.R.C.P., M.R.C.S., has been appointed clinical assistant in the skin department; J. R. Harper, L.R.C.P., M.R.C.S., has been appointed senior obstetric clerk; A. King, L.R.C.P., M.R.C.S., has been appointed resident house physician; A. C. Lankester, L.R.C.P., M.R.C.S., has been reappointed house surgeon; H. Low, M.A., M.B., B.S.Cantab, L.R.C.P., M.R.C.S., has been appointed resident accoucher; W. H. Millar, L.R.C.P., M.R.C.S., has been appointed clinical assistant in the throat department; W. H. Nix, B.A.Cantab, has been reappointed house surgeon; L. A. J. Rouillard, M.B., B.C.Cantab, L.R.C.P., M.R.C.S., has been appointed assistant house surgeon; A. W. F. Sayres, L.R.C.P., M.R.C.S., has been appointed clinical assistant in the ear department; E. F. Shearer, B.A., B.M., B.C. Oxon, has been appointed assistant house surgeon; A. F. Stabb, L.R.C.P., M.R.C.S., has been reappointed house surgeon; W. F. Umney, L.R.C.P., M.R.C.S., has been appointed resident house physician; E. E. Ware, L.R.C.P., M.R.C.S., has been reappointed house surgeon; G. H. Wickham, B.A., M.B., B.C.Cantab, L.R.C.P., M.R.C.S., has been reappointed non-resident house physician. At St. Mary's Hospital, James Cagney, M.A., M.D., M.CH.M.A.O.Roy.Univer., has been appointed electro-therapeutical officer. At the City of London Hospital for Diseases of the Chest, Victoria Park, A. Gaster, M.D. Bucharest, L.R.C.P.Lond., has been appointed house physician. At the City of London Lunatic Asylum, near Dartford, Algernon Wilson Lyons, M.B., L.R.C.P.Lond., M.R.C.S., of King's College, London, has been appointed resident clinical assistant. At the Royal Berks, Hospital, Reading, Ernest Ward, M.R.C.S., L.R.C.P., has been appointed house surgeon. At the Hospital, King's Lynn, Bertram W. Hogarth, M.R.C.S., L.R.C.P., has been appointed house surgeon and secretary.

### STUDENTS' MEDICAL SOCIETIES.

### Royal Veterinary College Medical Association.

The 137th ordinary general meeting was held in the theatre of the College on Wednesday, November 26th, 1890, at half-past six p.m., Mr. A. Smith (vice-president) in the chair. There were present the President, Professor Axe, forty-one student members, nine visitors, and the assistant secretary. The minutes of the last meeting were read and confirmed. Mr. H. G. Westgate first introduced a most interesting specimen of "Hydrocephalus" in the head of a calf. The case was explained by the President. The adjourned discussion on Mr. H. Session's paper on "Some Diseases of Calves" was resumed, but as few questions were forthcoming, a hearty vote of thanks was proposed, seconded, and carried, to the essayist for his interesting paper. Mr. J. H. Taylor was next called upon to read his paper on "The Diseases of the Horse's Foot." He commenced his paper by pointing out the importance of the subject, and roughly ran over the anatomy of the foot, the diseases affecting the bones and cartilages, and then those originating in the horn-secreting structures, and, lastly, those produced by injuries were in turn discussed, together with their causes, symptoms, and treatment. A lively discussion took place, and various questions were asked, the chief points of discussion being in regard to the use of hot or cold fomentations in laminitis, the cause of the descent of the pedal bone in the same disease, the hereditary predisposition question in relation to the side bones, and the treatment of canker. A vote of thanks to the essayist for his paper, and to the Chairman for presiding, brought the proceedings to a close.

### NOTES FROM THE SCHOOLS.

### CAMBRIDGE UNIVERSITY.

Performance of the "Ion."—The "Ion" of Euripides was given with great success by the Cambridge undergraduates. The performance went without a hitch of any kind, and the incidental music, the work of Mr. Charles Wood, Mus.B., was much appreciated.

Increased Examination Fees.—It is announced that the fee for each part of each of the three M.B. examinations is to be raised to ten guineas.

State Medicine.—There were 57 candidates for the last examination of diploma in public health. Of these, 41 received diplomas.

### OXFORD UNIVERSITY.

Mr. Frederick Howard, B.A., Balliol College, has been elected to the Burdett-Coutts Scholarship in Geology.

There will be an examination for a studentship in chemistry at Christchurch on January 13th.

At Balliol College, C. E. West, Merchant Taylors' School, Crosby, has been elected to the Brackenbury Science Scholarship.

At Christ Church College, to a scholarship in natural science, Marsh Hesketh, High School, Newcastle-under-Lyme; to a college exhibition in natural science, Arthur Langford Still, Grammar School, Tonbridge.

The Oxford University Calendar for 1891 is just published. There appear to have been fewer than usual changes in the academic staff. Professor Ray Lankester appears as Deputy Professor of Human and Comparative Anatomy; and the lamented death of Professor Rogers leaves a vacancy yet unfilled in the chair of Political Economy. From the index at the end of the volume, compared with that of 1890, we gather that the members of the University are at present stationary. There were 3,145 undergraduates on the books in the 1890 Calendar, and in the present 3,110. In 1888 the matriculations were 772, as compared with 787 in 1889. In 1888, 381 took the M.A. degree and 619 the B.A. degree, while in 1889 the Masters of Arts were 375 and the Bachelors of Arts 620. The University of the Punjab appears to be the one additional institution which has been affiliated to the University during the past year.

### EDINBURGH UNIVERSITY.

Students' Representative Council.—Office-bearers for the year 1890-91: Presidents, L. C. D. Douglas, D. H. Bugling, B.A., Donald G. Campbell; Secretaries, D. B. Boyle and W. Lyall Wilson; Executive Committee:—Divinity, I. R. Fram; Law, R. Munro and C. B. Gillies-Smith; Medicine, P. C. Faithfull, J. N. Craig, T. G. Macormack, and R. A. Heath; Arts, R. S. Craig, J. Erskine Dods, and R. Burns-Begg.

The first "Smoker" got up by the Edinburgh University Hare and Hounds—a young but vigorous branch of the Athletic Club—was held in the Continental Hotel on the evening of Saturday, the 22nd inst., and was a most decided success. The varied and excellent programme could not fail to attract a large number of students, the hall, in fact, being inconveniently crowded. The greatest good-humour and good-fellowship prevailed, and encores were the order of the evening. Mention might be made of songs by the very popular Dr. Hepburn and by Messrs. Stoddart Walker, Eric Yeo, Peart-Thomas, A. McFadden, and Duckworth. Mr. Ehrke's violin solo was

**Notes from the Schools.**—(*Continued.*)

delightfully rendered and enthusiastically encored, while "The Dodos" with their banjos made a most welcome appearance, and in one song the outstanding peculiarities of "The Professors" were most humorously hit off to the evident delight of the company. At the piano Messrs. Pechell and Lowe were invaluable. Several toasts were proposed and enthusiastically drunk, to Dr. Hepburn; to Pollard and Joyce, the first and second of that days' two miles handicap; to the Edinburgh and Northern Harriers; to the Edinburgh University Hare and Hounds, whose energetic secretary, Mr. Green, in replying, thought that these pleasant gatherings might take place weekly, or at least much more frequently than they do, if only the other sections of the Athletic Club would bestir themselves. There is an evident abundance of musical talent in the ranks of the students, and a series of such enjoyable smokers could surely be arranged. The general opinion is that they should in future take place in the Hall of the Union, and after Saturday evening's experience there can be no doubt of their success.

### PASS LISTS.

### Royal College of Surgeons of England.

The following gentlemen, having passed the necessary examinations and having conformed to the bye-laws and regulations, were, at an ordinary meeting of the council on Thursday, the 13th inst., admitted members of the College, viz.:—

A. M. Gossage, L.R.C.P. Lond.. Oxford University and Westminster Hospital; A. W. Gray, L.K.Q.C.P.I., Birmingham General Infirmary; W. S. Griffiths, L.R.C.P. Lond., St. Thomas's Hospital; J. F. Gummow, L.R.C.P. Lond., Charing Cross Hospital; J. B. Hall, L.R.C.P. Lond.. Cambridge University and Leeds General Infirmary; R. Hamilton, L.R.C.P. Lond., Manchester Royal Infirmary; J. R. Harper, L.R.C.P. Lond., St. Thomas's Hospital; C. S. Hawkes, L.R.C.P. Lond., London Hospital; T. I. Henning, L.R.C.P. Lond., Galway and St. Bartholomew's Hospital; W. H. Henshaw, L.R.C.P. Lond., Manchester Royal Infirmary; W. Hichens, L.R.C.P. Lond., London Hospital; T. F. Higgins, L.K.Q.C.P.I., Manchester Royal Infirmary; R. H. Holt, L.R.C.P. Lond., St. Mary's Hospital; W. Horsfield, L.R.C.P. Lond., Manchester Royal Infirmary; G. Y. C. Hunter, L.R.C.P. Lond., St. George's Hospital; H. S. Jackson, L.R.C.P. Lond., Bristol Royal Infirmary; H. W. John, L.R.C.P. Lond., Guy's Hospital; D. J. Jones, L.R.C.P. Lond., Charing Cross Hospital; W. J. C. Keats, L.R.C.P. Lond., St. Bartholomew's Hospital; C. I. Kirton, L.R.C.P. Lond., London Hospital; G. H. Knapp, L.R.C.P. Lond., Guy's Hospital; W. H. Lendrum, M.D.Q.U. Ireland, Belfast Royal Hospital; C. P. Le Quesne, L.R.C.P. Lond., St. Bartholomew's Hospital; G. D. B. Levick, L.R.C.P. London, Middlesex Hospital; R. M. Littler, L.R.C.P. Lond., Manchester Royal Infirmary; G. Lys, L.R.C.P. Lond., London Hospital; A. E. Mahood, M.B.R.U. Ireland, Dublin and Birmingham General Hospital; H. F. Mantell, L.R.C.P. Lond.. St. Mary's Hospital; H. Mason, L.R.C.P. Lond., General Hospital, Birmingham; S. F. Mawson, L.R.C.P. Lond., Manchester Royal Infirmary; H. F. Mole, L.R.C.P. Lond., Bristol Royal Infirmary; J. E. Molson, L.R.C.P. Lond., Middlesex Hospital; M. S. F. Monier-Williams, L.R.C.P. Lond., St. George's Hospital; E. M. Morphew, L.R.C.P. Lond., University College Hospital; W. B. Morton, L.R.C.P. Lond., University College Hospital; H. A. de Beauvoir-Nelson, L.R.C.P. Lond., St. Bartholomew's Hospital; W. T. Ohlmüs, L.R.C.P. Lond., Ceylon Medical College; J. Panting, L.R.C.P. Lond., Combridge University and Middlesex Hospital; T. L. Pennell, L.R.C.P. Lond., University College Hospital; C. S. Pethick, L.R.C.P. Lond., St. Bartholomew's Hospital; H. M'Donald Phillpotts, L.R.C.P. Lond., St. Mary's Hospital; H. W. Pooler, L.R.C.P. Lond., Queen's Hospital, Birmingham; F. B. Reilly, L.R.C.P, Lond., Charing Cross Hospital; G. L. H. Revill, L.R.C.P. Lond., Charing Cross Hospital; H. Rhodes, M.B. Glasgow, Glasgow University; N. L. Richards, L.R.C.P. Lond., Guy's Hospital; R. W. Richards, L.R.C.P. Lond., St. Bartholomew's Hospital; L. Roberts, L.R.C.P. Lond., St. Bartholomew's Hospital; A. W. F. Sayres, L.R.C.P. Lond., St. Thomas's Hospital; J. R. Scott, L.R.C.P. Lond., St. Thomas's Hospital; J. H. Shaw. L.R.C.P. Lond., Royal Infirmary, Liverpool; W. R. Shortt, L.R.C.P. Lond., St. George's Hospital; H. A. Smith, L.R.C.P. Lond., Guy's Hospital; O. A. Stott, L.R.C.P. Lond., Yorks. College and General Infirmary, Leeds; R. Stuart, L.R.C.P. Lond., St. George's Hospital; H. Tempest, L.R.C.P. Lond., Yorkshire College and General Infirmary, Leeds; H. J. Trizard, L.R.C.P. Lond., St. George's Hospital; W. F. Umney, L.R.C.P. Lond., St. Thomas's Hospital; C. M. Vernon, L.R.C.P. Lond., Bristol Royal Infirmary; L. Wainwright, L.R.C.P. Lond., London Hospital; R. M. Hodson Walford, L.R.C.P. Lond., St. George's Hospital; K. S. Walfridsson, L.R.C.P. Lond., London Hospital; A. J. Williams, L.R.C.P. Lond., King's College Hospital; C. W. Wilson, M. D. M'Gill, M'Gill College, Montreal; J. G. Wilson, L.R.C.P. Lond., London Hospital; G. M. Winter, L.R.C.P. Lond., St. Mary's Hospital; G. E. M. Wood, L.R.C.P. Lond., University College Hospital; P. Worley, L.R.C.P. Lond., Manchester Royal Infirmary; and S. Zeidan, L.R.C.P. Lond., St. Thomas's Hospital.

### London B.Sc. Examination.

FIRST DIVISION.—B. L. Abrahams, University College; Walter Aston, B.A., Birkbeck Institution; Richard William Bathe, Birkbeck Institution; Arthur Seal Blackwell, St. Bartholomew's Hospital; Horace Edward Brothers; Edwin Budden; James Alfred

**The Hospital Advertiser.**

**Pass Lists—(*continued*).**

Butterfield; Thomas Cartwright, B.A.; James Stansfield Collier, St. Mary's Hospital, City Guilds; Harry Edwin Hadley; Edward M. Hainworth, St. Thomas's Hospital and Birkbeck Institution; James Hendrick, King's College and Birkbeck Institution; John Theodore Hewitt, Hartley Institution, Normal School of Science, and St. John's College, Cambridge; Frederick Gowland Hopkins, Guy's Hospital; Owen Glynne Jones, City Guilds Central Institute; Duncan Scott Macnair, Owens College and University, Würzburg; Thomas Goddard Nicholson, St. Thomas's Hospital; John Herbert Parsons, University Colleges, Bristol and London; Alfred William Porter, University Colleges, Liverpool and London; Herbert Harold Robjohns, University College, Aberystwith; John Henry Salter, Dalton Hall and Owens College; George Adolphus Schott, Trinity College, Cambridge; John Stephenson, Owens College; James Floyd Tristram; Gilbert Thomas Walker, Trinity College, Cambridge; William Watson, Normal School of Science; Arthur Willey, University College; William Bamford Winston, St. Thomas's Hospital and Birkbeck Institution.

Second Division.—Charles William Andrews, B.A., Birkbeck Institution; Lucy Baker, University College, Cardiff; Alfred Barker, M.A.; Landen Hall Barker, St. Mark's College, Chelsea; Frank Bastow, B.A.; Edward Jeremiah Bles, Owens College; Florence Buchanan, University College and London School of Medicine; John Le Mare Bunch, University College; Alfred Byers, Owens College; George Herbert Carpenter, King's College and Royal College of Science, Dublin; Frederick Daniel Chattaway, Christ Church, Oxford; Arthur Harry Church, University College, Aberystwith; William Ernest Dalby, Owens College; Gilbert Howard Daniel; John Smalley Dockray, Owens College; Percy James Edmunds, University College; Florence Gibson, Firth College; Lillah K. Given-Wilson, University College; Ernest William Hey Groves, University Colleges, Bristol and London; Harry Slater Holmes, Yorkshire College; George Thomas Horsley, Durham College of Physical Science; Annie Jane Hoyles, Firth College; William Fraser Hume, Normal School of Science; Arthur R. Hall Ingram, Mason College; Francis Oscar Lane; Sarah Beatrice Lowe, Westfield and University Colleges; John William Martin, Birkbeck Institution, Bromley School of Science; Thomas Percy Nunn, University College, Bristol; Martin J. O'Flanagan, Owens College; Maria Matilda Ogilvie, University College; Charles Frederick Rea, B.A., King's College; Frederick William Short, Pharmaceutical Society and Birkbeck Institution; Arthur Thomas Simmons, Normal School of Science; John Stretton Sloane, St. Bartholomew's Hospital; William Sydney Smith, University College, Nottingham; Ernest Jackson Smyth, University College and Birkbeck Institution; James Cerridfryn Thomas, University College, Aberystwith; Walter Thorp, Yorkshire and University Colleges; John Wade, University College; Herbert George Wells, Normal School of Science; Martha Annie Whiteley, Royal Holloway College; Walter Percy Winter, University College, Aberystwith; Claude Henry C. Woodhouse, Normal School of Science.

**ATHLETICS.—Football.**

Association Rules.—On November 25th.

The match between *Cambridge University* and the *Blackburn Rovers* resulted in a tie of two goals each.

The draw for the London Challenge Cup took place on the evening of the 25th at the offices of the Association, 61, Chancery Lane. The first-named clubs have choice of ground, and the round is to be decided on December 13. Appended are the ties: *Old Harrovians* v. *Clapton*; *Old St. Stephen's* or *Grove Park* v. *City Ramblers* or *Kildare*; *Millwall Athletic* v. *Chiswick Park*; *London Caledonians* v. *Casuals*; *Woodville* v. *St. Bartholomew's Hospital*; *Bowes Park* or *Barnes* v. *Tottenham Hotspur*; *Old St. Mark's* v. *Ilford*; *Old Westminsters* v. *Royal Arsenal*.

November 26th.

*King's College Hospital v. Clapham Rovers.*—This match was played on the Medicos' ground at Wormwood Scrubbs. The weather was bitterly cold and the ground very heavy. Sanderson kicked off for the visitors. The home side having won the toss, chose the railway goal. Some fast play resulted in mid-field, and Turner and Brett broke away, but were repelled by Ingram. Slaughter next obtained possession from a pass by Critchley, and sent in a low, swift shot, thus scoring the first goal for the Rovers. Shortly afterwards Tanner, with a good shot, equalised the score. In the second half of the game the play was of a very spirited nature, and soon Brett placed the Medicals a goal ahead. Slaughter and Sanderson each shot over the cross-bar. After this the ball was transferred to the opposite end of the ground, and Browning had to use his hands. Although some fast play followed, nothing more was added to the score, and King's won a well-gained victory by two goals to one. Sides: King's.—J. A. Procter (goal), E. Timis and H. W. Lyle (backs), C. Wace (captain), W. B. Bell, and E. G. Stores (half-backs), R. T. Turner and H. C. Brett (right), C. F. Backhouse (centre), E. A. Head and G. M. Hetherington (left). Clapham Rovers.—H. Browning (goal), W. Ingram and E. Critchley (backs), S. J. Northcote, C. Thompson, and T. P. Taylor (half-backs), W. Toomes and S. Colman (captain) (right), W. D. Sanderson (centre), E. Slaughter and A. C. Keeley (left).

*Royal Military Academy v. St. Mary's Hospital.*—This match, which was played at Charlton Park, resulted in a win for the Cadets by two goals to one. The game was started by Summerhayes, who set the ball in motion for the Medicals, who, having the wind in their favour, attacked in force. After some play, Rajoelson took the ball up the ground, and obtained the first goal for the Academy. The visitors then played up in fine style, and Montgomery had some work to do, but was equal to the occasion, and saved in good style, nothing further occurring up to half-time. After changing sides, Macgowan started the ball, which was soon got into the home territory, and Symes sent in a shot and equalised the score. Both sides now played up well, and Macgowan scored by a fine shot for the Academy. The remainder of the game being completed in semi-darkness no further score resulted. Sides:—R.M.A.: A. Montgomery (goal), C. Evans and S. F. Gosling (backs), W. H. Kay,

### Athletics—*continued.*

V. de V. Hunt, and H. L. Pritchard (half-backs), A. E. Turner (captain) and H. Rajoelson (left wing), G. L. Macgowan (centre), and E. H. Harper and E. G. Carbutt (right wing). St. Mary's Hospital: A. E. Drew (goal), E. G. Moon (captain) and E. P. Staples (backs), W. S. Patterson, B. Parer, and C. D. Lindsey (half-backs), E. W. Beesley and H. H. Francis (right wing), G. W. Summerhays (centre), E. W. Joserlyne and J. O. Symes.

RUGBY RULES—NOVEMBER 24TH.

A *Cambridge University Team* defeated a *Fifteen* got together by Mr. Carpmael, at Cambridge, by five goals to a goal and a try.

NOVEMBER 25TH.

*Cambridge University v. Edinburgh Academicals.*—This match was played at Cambridge in wintry weather. Forward Cambridge was weaker than at any period this season; their backs, as usual, were good. C. A. Hooper brought off one grand dodging run which fairly took the audience by storm. The visitors played a sterling game, especially F. B. Marjoribanks, H. F. Cadell, and W. F. Ferguson. The Edinburgh Academicals kicked off from the Selwyn end and at once forced the game into the University half; seven minutes from the start J. Duncan crossed the line, but was tackled by G. M'Gregor, the visitor getting the try. On changing ends the visitors had scored one try to nothing. The Scotsmen at once caused the Light Blues to act on the defensive. G. M'Gregor punted the ball into the visitors' twenty-five, where it remained for a time. It was next dribbled back close to the University goal-line, and from some loose play H. F. Cadell managed to get in. The home team now appeared to pull themselves together. C. A. Hooper made a magnificent run. P. H. Morrison ran grandly and was pushed over the boundary. Nothing further was scored, and the Academicals were thus the victors by two tries to nothing. R. Hontgomery (Trinity College), and C. Chernside (Edinburgh Academicals), touch judges; W. S. Goodwin (Northampton), referee.

*Oxford University v. Midland Counties.*—This match was played at Oxford in bitterly cold weather, and a sharp frost overnight had made the ground very hard. The visitors were not well represented, and were defeated by four tries and four touches down to nil. The game for the first quarter of an hour was fairly even, but following a good bit of play on the part of North, Paterson, and J. H. G. Wilson, the latter crossed the line, the attempt of goal by Fleming being unsuccessful. At this point snow began to fall, and the play was consequently affected to a great extent. The University had the best of it, and Parkins gained a second try for them. The Dark Blues' superiority was very apparent. Slater, Morris, and Deykin tried hard to stem the tide, but S. E. Wilson pierced their ranks and obtained a try. The game was an uninteresting one, the Oxonians being superior at all points. Percival, Kay, North, Morris, Dicker, and Rogers among the forwards, and De Winton, Cochrane, and Hill behind, showed good form for their respective clubs.

NOVEMBER 26TH.

*East Sheen v. Guy's Hospital.*—This match was played on the Richmond Athletic Association Ground, and resulted in a drawn game of three tries each. Guy's starting with the wind behind them, H. Cooper and J. J. E. Biggs each got in, the latter failing with the place kick. After changing ends, A. Bromet, with a good dribble, gained a try, which H. J. W. Lovelace just failed to convert, Biggs touching the ball as it was passing over the bar. After this Brenchley got in, but Lovelace failed with the place kick, and J. H. Bettington scored for Guy's. Reid not converting, there followed some forward play close to the hospital goal, and Bromet made the score even, at which it remained, Lovelace's place kick being a failure.

*University College Hospital v. Royal Veterinary College.*—This match was played at Park, Tottenham, and resulted in a victory for U.C.H. by one goal (dropped by T. D. Bell) and a try by P. H. Stirk to one try by Edwards. The Hospital played a man short. Teams: U.C.H.—Birks (captain) (back); Stokes, Ball, and Griffiths (three-quarter backs); Miles and Davies (half-backs); Martin, Stirk, Gubbin, R. Bartlett, Blenkinsop, T. Bartlett, Crosby, and Abrams (forwards). R.V.C.—Hodge (back); Jones, Case, Marriott (three-quarter backs); Harris (captain) and Harvey (half-backs); Horsley, Ball, Bowker, Master, Williams, James, Davies, Edwards, Robertson (forwards).

FATAL FOOTBALL ACCIDENT.—Mr. H. M. Walters, brother to P. M. and A. M. Walters, the well-known International football players, has died from the effects of a kick in the abdomen, received on November 12th, while playing for the Casuals' Association team against St. Bartholomew's Hospital. Peritonitis rapidly followed the injury, and he gradually became worse and died. The accused was educated, first at Haileybury School, and then at Oriel College, Oxford. He received his blue and played against Cambridge in 1889. His death has given a great shock to the football world, and several matches which were to have been played last Saturday were postponed.

### Edinburgh University Hare and Hounds.

A two-mile handicap race of this club was held on the 22nd. The weather was bad, there being a strong wind and rain. Pollard and Joyce, both scratch, were first and second. Time, 11 min. 2 sec.

### EVENTS FOR THE MONTH OF DECEMBER.

[Items intended for this column must reach the office, 140, Strand, W.C., not later than first post on Monday mornings.]

**December Meetings and Athletics.**

For list of Students' Medical Societies' Meetings and Football Matches during December, see THE HOSPITAL for Nov. 29ht.

